# *Gynura procumbens* (Lour.) Merr. and Its Active Compound Attenuate Oleic Acid-Induced Hepatocyte Steatosis and Modulate Cholesterol Metabolism-Related Gene Expression

**DOI:** 10.3390/molecules31152708

**Published:** 2026-08-04

**Authors:** Kimberlyn Feguro, Norsyahida Mohd Fauzi, Khairana Husain, Sarmila Hanim Mustafa, Malina Jasamai

**Affiliations:** 1 Centre for Drug and Herbal Development, Faculty of Pharmacy, Universiti Kebangsaan Malaysia, Kuala Lumpur 50300, Malaysia; kfeguro@usa.edu.ph (K.F.); khairana@ukm.edu.my (K.H.); sarmila.hanim@gmail.com (S.H.M.); malina@ukm.edu.my (M.J.); 2 College of Pharmacy and Medical Technology, University of San Agustin, Iloilo City 5000, Philippines

**Keywords:** MASLD, *Gynura procumbens*, stigmasterol, SREBP2, PCSK9

## Abstract

Metabolic dysfunction-associated steatotic liver disease (MASLD) is characterised by excessive lipid accumulation in hepatocytes and often accompanied by dyslipidaemia. *Gynura procumbens*, a medicinal plant native to Southeast Asia, exhibits lipid-lowering and hepatoprotective properties. However, its effects on hepatic lipid accumulation and cholesterol metabolism-related gene regulation remain unclear. This study evaluated the effects of *G. procumbens* extract (GPCE) and stigmasterol on oleic acid-induced hepatocyte steatosis and cholesterol metabolism-related gene expression. GPCE was assessed for HMG-CoA reductase (HMGCR) inhibition, lipid accumulation, and expression of cholesterol metabolism-related genes (*HMGCR*, *SREBP2*, *LDLR*, and *PCSK9*). Bioassay-guided fractionation identified stigmasterol as a constituent of the highly active fractions and its structure was characterised by LC-TOF-MS and NMR. GPCE and stigmasterol inhibited HMGCR activity by 86.31 ± 5.3% and 56.59 ± 7.6%, respectively. Both treatments reduced lipid accumulation in hepatocytes and modulated the mRNA expression of selected cholesterol homeostasis-related genes, including transcriptional downregulation of *SREBP2* and *PCSK9*. These findings suggest that *G. procumbens* possesses lipid-lowering activity and that stigmasterol may represent one of the bioactive constituents contributing to its observed biological effects. Further studies involving protein level validation, functional assays and in vivo studies are warranted to fully elucidate the mechanisms and establish the biological relevance of these findings in MASLD.

## 1. Introduction

Metabolic dysfunction-associated steatotic liver disease (MASLD) is the leading cause of chronic liver disease worldwide, with an estimated global prevalence of 30% [1]. The disease spectrum ranges from simple hepatic steatosis to metabolic dysfunction-associated steatohepatitis (MASH), which is characterised by hepatic fat accumulation, inflammation and fibrosis [2,3]. At cellular level, hepatic steatosis in MASLD arises from an imbalance between de novo lipogenesis, fatty acid β-oxidation, lipid export and sterol metabolism, leading to the accumulation of triglycerides in hepatocytes [4]. Although excessive triglyceride accumulation is the hallmark of hepatic steatosis, dysregulated cholesterol homeostasis, driven by imbalances in cholesterol synthesis, uptake, and regulation, also contributes to the MASLD pathogenesis and disease progression [5,6]. Consequently, steatosis in MASLD is associated with changes in key genes involved in cholesterol homeostasis including sterol regulatory element-binding protein 2 (SREBP2), 3-hydroxy-3-methylglutaryl-CoA reductase (HMGCR), low-density lipoprotein receptor (LDLR), and proprotein convertase subtilisin/kexin type 9 (PCSK9), which play important roles in maintaining hepatic cholesterol balance [6].

*Gynura procumbens* (Lour.) Merr. (Family Asteraceae) is a small green herbal plant that is found in tropical Asian countries, including Malaysia. Extracts of *G. procumbens* exhibits antihyperglycaemic [7,8], antioxidant [9,10], anti-inflammatory [11,12], and antihyperlipidaemic activities [13,14]. Phytochemical investigations have identified a broad spectrum of bioactive constituents in *G. procumbens*, including phenolic acids and flavonoids such as gallic acid, chlorogenic acid, kaempferol, and quinic acid as well as volatile terpenes like α-pinene and limonene [10,11,12,15]. Among these, trans-*p*-coumaric acid has been shown to reduce hepatic lipid accumulation in an alcoholic-induced fatty liver disease rodent model [16]. Additionally, *G. procumbens* contains stigmasterol [11,17]. Due to its structural similarity to cholesterol [18], stigmasterol has the potential to modulate sterol-related metabolic pathways. However, its effects on hepatocellular lipid accumulation and cholesterol homeostasis-related gene expression remain poorly understood.

Although several animal studies have demonstrated that *G. procumbens* extract and stigmasterol can ameliorate steatosis driven by alcohol or metabolic dysfunction [15,19,20,21], evidence regarding their effects on hepatocellular cholesterol homeostasis, particularly in MASLD, remains limited. It is unclear whether *G. procumbens* and stigmasterol can coordinate the expression of cholesterol-related genes such as SREBP2, HMGCR, LDLR and PCSK9 in lipid-overloaded hepatocytes. To investigate these hepatocellular responses, human-derived hepatocyte cell lines and advanced culture systems are commonly employed. Human hepatoma HepG2 cells are an established model for studying hepatic lipid metabolism and cholesterol regulatory pathways, as they retain key metabolic regulatory mechanisms despite their tumour origin [22,23]. Exposing HepG2 cells to oleic acid (OA) is widely used as a reproducible in vitro model to induce pronounced intracellular lipid accumulation, providing a reliable platform to evaluate downstream alterations in lipid and cholesterol homeostasis-related gene expression including genes central to cholesterol homeostasis [22]. Although OA-induced HepG2 steatosis does not fully capture the complexity of MASLD, it offers a controlled model to investigate early changes in hepatocellular lipid handling and associated metabolic responses.

We hypothesised that *G. procumbens* extract and stigmasterol attenuate OA-induced lipid accumulation and that these effects may be accompanied by alterations in cholesterol homeostasis-related gene expression in hepatocytes. Therefore, this study aimed to investigate the effects of hydroethanolic *G. procumbens* crude extract (GPCE) and stigmasterol on lipid accumulation and associated changes in cholesterol homeostasis-related gene expression in an OA-induced hepatocyte steatosis model.

## 2. Results

### 2.1. Inhibitory Effect of G. procumbens Extract and Fractions on HMGCR Activity

**Figure 1:**
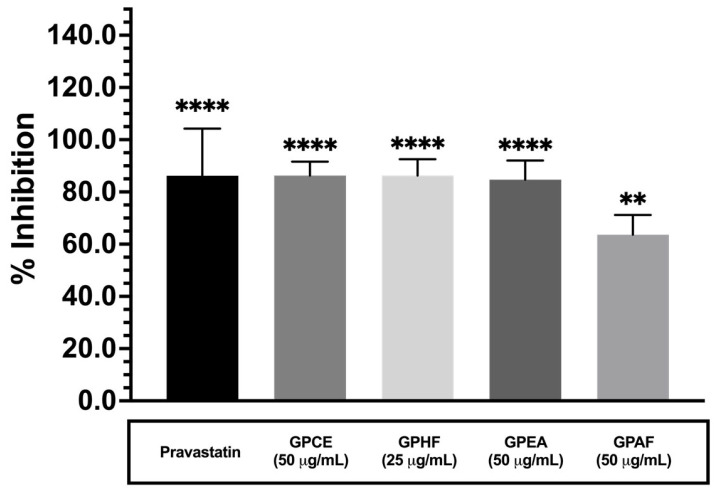
*G. procumbens* extract and fractions inhibit HMGCR activity in vitro. The inhibitory effects of GPCE (50 μg/mL), hexane fraction (GPHF, 25 μg/mL), ethyl acetate fraction (GPEA, 50 μg/mL), and acetone fraction (GPAF, 50 μg/mL) were evaluated using an in vitro HMGCR enzymatic assay. Pravastatin (2 μM) was used as positive control. Data are presented as mean ± SEM from three independent experiments (*n* = 3). ** *p* < 0.01, **** *p* < 0.0001 versus control.

The crude extract (GPCE) inhibited HMG-CoA reductase activity by 86.31 ± 5.3%. Among the fractions, the hexane fraction (GPHF) showed the highest inhibition (86.28 ± 6.5%) followed by the ethyl acetate fraction (GPEA, 84.72 ± 7.3%), while the acetone fraction (GPAF) exhibited the lowest inhibitory effect (63.7 ± 7.5%). The kit-provided positive control, pravastatin, inhibited the enzyme by 86.3 ± 18.0% under the same conditions (Figure 1).

### 2.2. Effect of G. procumbens Extract and Fractions on HepG2 Cell Viability

**Figure 2:**
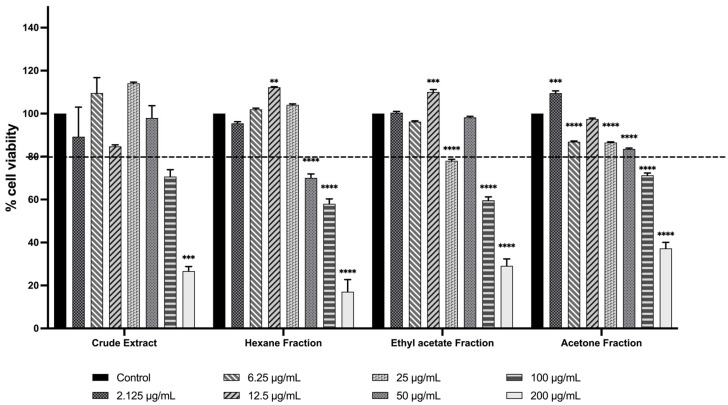
Effect of *G. procumbens* extract and fractions on HepG2 cell viability. HepG2 cells were treated for 24 h with different concentrations of GPCE and solvent fractions including GPHF, GPEA and GPAF, and cell viability was assessed. Concentrations that maintained cell viability at ≥80% were selected for subsequent experiments. Data are presented as mean ± SEM from three independent experiments (*n* = 3). Statistical significance is indicated as ** *p* < 0.01, *** *p* < 0.001, and **** *p* < 0.0001 versus untreated control.

Figure 2 shows that the GPCE, GPHF, GPEA and GPAF maintained ≥80% HepG2 cell viability at lower concentrations (≤25 µg/mL). At 50 µg/mL, GPHF reduced cell viability to below 80%, whereas GPCE and other fractions maintained cell viability above 80%. In contrast, treatment with extract and fractions at higher concentrations (100 and 200 µg/mL) resulted in a reduction of cell viability below 80%. Only concentrations that maintained ≥80% cell viability were selected for subsequent experiments.

### 2.3. Effect of G. procumbens Extract and Fractions on OA-Induced Lipid Accumulation and Cholesterol-Regulating Gene Expression in HepG2 Cells

**Figure 3:**
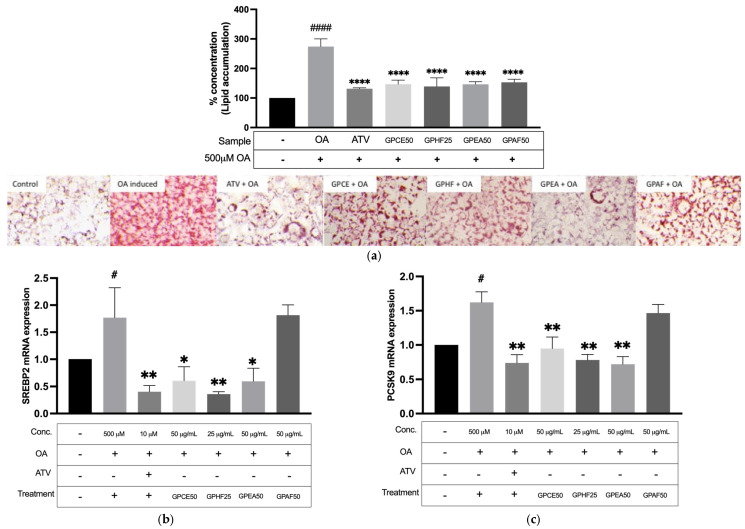
Effect of *G. procumbens* crude extract and fractions on OA-induced lipid accumulation and cholesterol-regulating gene expression in HepG2 cells. (**a**) Quantitative intracellular lipid accumulation (%) measured via Oil Red O (ORO) spectrophotometric analysis, accompanied by representative ORO staining microscopy images (100× magnification). Control cells were maintained in standard medium; the model group induced with 0.5 mM OA for 24 h; treatment groups were pretreated with either atorvastatin or extract and fractions for 12 h prior to OA induction. Time course mRNA expression levels of (**b**) *SREBP2* and (**c**) *PCSK9*. Data are presented as mean ± SEM (*n* = 3 biological replicates). Statistical significance is indicated as * *p* < 0.05, ** *p* < 0.01, and **** *p* < 0.0001 versus OA-treated cells, and *^#^ p* < 0.05 and *^####^ p* < 0.0001 versus untreated control.

As shown in Figure 3a, pretreatment of HepG2 cells with GPCE markedly reduced OA-induced intracellular lipid accumulation compared with OA-treated cells. Similar lipid-lowering effects were observed for the hexane (GPHF) and ethyl acetate (GPEA) fractions, whereas the acetone fraction (GPAF) showed a weaker effect. To further elucidate the underlying molecular mechanisms, the effects of *G. procumbens* extract and its fractions on the expression of cholesterol-regulating genes were evaluated. Pretreatment with GPCE, GPHF and GPEA significantly downregulated the mRNA expression of *SREBP2* (Figure 3b) and *PCSK9* (Figure 3c) compared with OA-induced cells. In contrast, GPAF did not inhibit the expression of either gene.

### 2.4. Isolation and Identification of Isolated Stigmasterol (Compound ***1***) from G. procumbens

**Figure 4:**
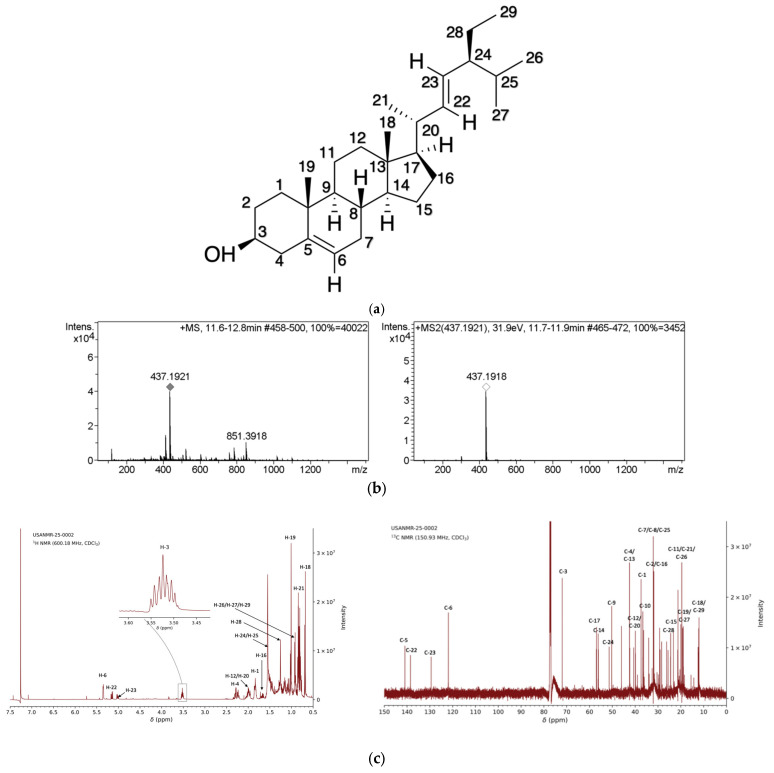
Compound **1** identified as stigmasterol. (**a**) Chemical structure of stigmasterol with carbon numbering used for NMR assignments. (**b**) LCMS and MS/MS spectra of compound HFSF1 in positive mode showed molecular ionisation at *m*/*z* 437.1918, consistent with a [M + Na]^+^ adduct. (**c**) ^1^H and ^13^C NMR spectra of the compound **1**.

Compound **1** was isolated from the ethanolic leaf extract of *G. procumbens* as a white amorphous powder (Rf ≈ 0.45 in hexane:ethyl acetate, 9:1; *v*/*v*; mp ≈ 162 °C). LC–MS/MS [electrospray ionisation (ESI), positive mode] showed an ion at *m*/*z* 437.1918 [M + Na]^+^, consistent with stigmasterol (MW 412.70) (Figure 4b). In Figure 4c, the ^1^H Nuclear Magnetic Resonance (NMR) spectrum displayed characteristic methyl signals (δ 0.72–0.92), a hydroxyl-bearing methine proton at δ 3.54 (H-3), and olefinic protons at δ 5.38 (H-6) and δ 5.04 and 5.18 corresponded to double bonds at the C-5/C-6 and C-22/C-23 positions. The ^13^C NMR spectrum confirmed 29 carbons, including olefinic carbons at δ 140.77 (C-5), 121.74 (C-6), 138.33 (C-22), and 129.28 (C-23), and six methyl carbons at δ 11.87 (C-18), 19.41 (C-19), 21.22 (C-21), 21.09 (C-26), 18.79 (C-27), and 12.26 (C-29). Overall, the MS and NMR data matched published values for stigmasterol [16,20,21], thereby confirming the identity of compound **1**.

### 2.5. Inhibitory Effect of Stigmasterol on HMG-CoA Reductase Activity

**Figure 5:**
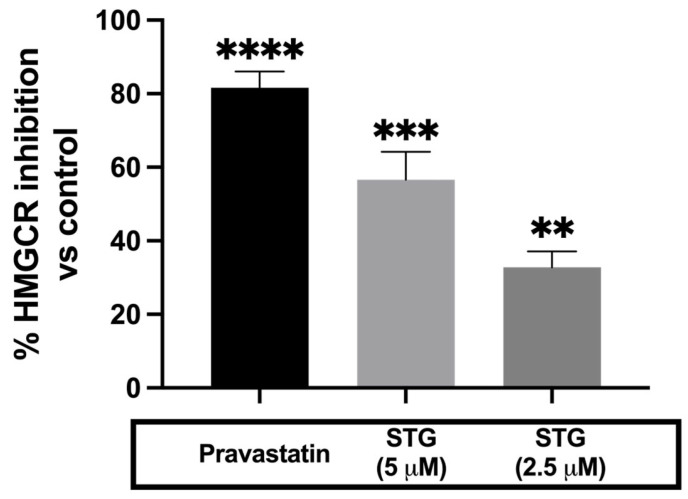
Stigmasterol inhibit HMG-CoA reductase activity in vitro. The inhibitory effects of stigmasterol (2.5 and 5 μM) were evaluated using an in vitro HMG-CoA reductase enzymatic assay. Pravastatin (2 μM) was used as a positive control. Data are presented as mean ± SEM from three independent experiments (*n* = 3). Statistical significance is indicated as ** *p* < 0.01, *** *p* < 0.0005, **** *p* < 0.0001 versus control.

Figure 5 shows that stigmasterol inhibited HMG-CoA reductase activity in a concentration-dependent manner with 32.79 ± 4.36% at 2.5 μM and 56.59 ± 7.61% at 5 μM. The kit-provided positive control, pravastatin, inhibited the enzyme by 80% under the same condition. Both stigmasterol concentrations showed significant inhibition versus control.

### 2.6. Effect of Stigmasterol on HepG2 Cell Viability

**Figure 6:**
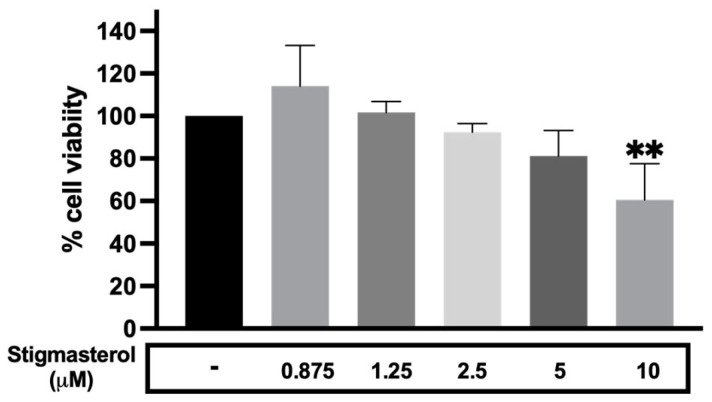
Effect of stigmasterol on HepG2 cell viability. HepG2 cells were treated for 24 h with different concentrations of stigmasterol, and cell viability was assessed. Concentrations that maintained ≥80% cell viability were selected for subsequent experiments. Data are presented as mean ± SEM from three independent experiments (*n* = 3). Statistical significance is indicated as ** *p* < 0.01 versus untreated control.

Figure 6 shows that stigmasterol maintained ≥80% HepG2 cell viability at concentrations up to 5 µM. Cell viability remained above the acceptable threshold at 0.875, 1.25, and 2.5 µM, and was still approximately ≥80% at 5 µM. In contrast, treatment with stigmasterol at the higher concentration (10 µM) reduced cell viability to below 80% (*p* < 0.01). Accordingly, only concentrations that maintained ≥80% cell viability were selected for subsequent experiments.

### 2.7. Effect of Stigmasterol on OA-Induced Lipid Accumulation and Cholesterol-Regulating Gene Expression in HepG2 Cells

**Figure 7:**
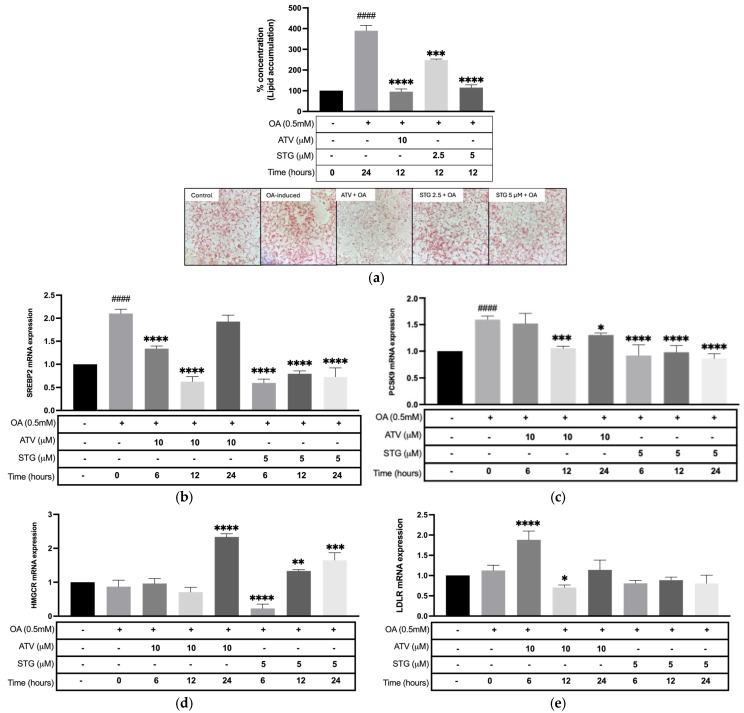
Effect of stigmasterol on OA-induced lipid accumulation and cholesterol homeostasis-related gene expression in HepG2 cells. (**a**) Quantitative intracellular lipid accumulation (%) measured via ORO spectrophotometric analysis, accompanied by representative ORO staining microscopy images (100× magnification). Control cells were maintained in standard medium; the model group induced with 0.5 mM OA for 24 h; treatment groups were pretreated with either atorvastatin or stigmasterol for 12 h prior to OA induction. Time course mRNA expression levels of (**b**) *SREBP2*, (**c**) *PCSK9*, (**d**) *HMGCR*, and (**e**) *LDLR*. Data are presented as mean ± SEM (*n* = 3 biological replicates). Statistical significance is indicated as * *p* < 0.05, ** *p* < 0.01, *** *p* < 0.001, and **** *p* < 0.0001 versus OA-treated cells, and *^####^ p* < 0.0001 versus untreated control.

Figure 7a demonstrates that pretreatment with stigmasterol significantly attenuated OA-induced intracellular lipid accumulation in HepG2 cells compared to the OA-treated cells. To determine whether this lipid-lowering effect was accompanied by changes in cholesterol homeostasis-related gene expression, the mRNA expression of *SREBP2*, *PCSK9*, *HMGCR* and *LDLR* was evaluated after 6, 12 and 24 h of treatment. Stigmasterol (5 µM) consistently reducing OA-induced *SREBP2* mRNA expression at all three timepoints (Figure 7b). In comparison, atorvastatin (10 µM) reduced *SREBP2* expression at 6 and 12 h, but this reduction was not sustained after 24 h. Similarly, stigmasterol significantly decreased *PCSK9* mRNA expression at all evaluated timepoints. In contrast, atorvastatin did not alter *PCSK9* expression at 6 h but exhibited significant inhibition at 12 and 24 h (Figure 7c). For *HMGCR*, no significant difference in mRNA expression was observed between OA-treated and untreated control cells (Figure 7d). Following stigmasterol treatment, HMGCR mRNA expression exhibited a time-dependent response, with a significant reduction at 6 h and increased expression at 12 and 24 h compared with the OA-treated group. In comparison, atorvastatin produced minimal changes in HMGCR expression during the first 12 h but significantly increased its expression after 24 h. For LDLR, no significant difference in mRNA expression was observed between OA-treated and untreated control cells (Figure 7e). Following stigmasterol treatment, LDLR mRNA expression remained unchanged at all evaluated time points compared with the OA-treated group. In contrast, atorvastatin (10 µM) induced a transient change in LDLR expression, with a significant increase observed at 6 h, followed by a significant reduction at 12 h, while no significant difference was detected after 24 h (Figure 7e). Overall, stigmasterol attenuated OA-induced lipid accumulation while consistently reducing OA-induced *SREBP2* and *PCSK9* mRNA expression across all time points. In contrast, its effects on *HMGCR* expression varied over time, while no significant changes were observed in *LDLR* expression.

## 3. Discussion

MASLD is a complex metabolic spectrum characterised by excessive hepatocellular lipid accumulation, altered lipid handling, and dysregulated systemic and cellular cholesterol homeostasis [4]. Although *G. procumbens* has been widely reported to exhibit lipid-lowering [13,14] and hepatoprotective activities in vivo [6,20], prior work has largely focused on systemic lipid outcomes or broader liver injury endpoints rather than directly interrogating hepatocellular lipid accumulation together with key cholesterol regulatory genes namely *HMGCR*, *SREBP2*, *LDLR*, and *PCSK9* in a MASLD-relevant in vitro steatosis model. Moreover, the extent to which specific bioactive constituents contribute to these effects remains insufficiently defined. To address these gaps, this study evaluated the effects of crude extract, its fractions, and stigmasterol on OA-induced hepatocyte steatosis model. Given that MASLD encompasses both hepatocellular lipid overload and disturbed cholesterol homeostasis [6,24], this study was designed to capture a functional anti-steatotic endpoint together with associated changes in cholesterol homeostasis-related gene expression.

We initiated our study with a cell-free HMGCR assay as a target-focused biochemical screen. HMGCR is the rate-limiting enzyme in cholesterol biosynthesis and a clinically validated target for dyslipidaemia [5,6], which is particularly relevant given that hepatic cholesterol accumulation contributes to lipotoxicity and MASLD progression [6,24]. Furthermore, because stigmasterol is a phytosterol structurally analogous to cholesterol [18], evaluating its direct interaction with core sterol-regulatory enzymes was a logical starting point. Although cell-free HMGCR inhibition does not directly assess triglyceride-driven steatosis, hepatic cholesterol synthesis and triglyceride metabolism are closely interconnected through shared regulatory networks such as SREBP2 [6,25]. Therefore, we progressed to an OA-induced steatotic HepG2 cells to determine whether the HMGCR inhibitory activity observed in the cell-free assay translated into reduced intracellular lipid accumulation and modulation of cholesterol homeostasis-related genes under conditions of fatty acid overload. Our results demonstrated that GPCE, as well as its hexane and ethyl acetate fractions, markedly attenuated OA-induced intracellular lipid accumulation. Together with its HMGCR inhibitory activity, these findings highlight the potential of *G. procumbens* to influence hepatic lipid metabolism. These in vitro findings build upon previous in vivo study demonstrating that *G. procumbens* reverses ethanol-induced liver steatosis [15] and alleviates MASH [20]; our study expands current understanding by demonstrating its ability to reduce lipid accumulation in an OA-induced hepatocellular steatosis model relevant to MASLD.

Guided by these biological activities, we successfully isolated stigmasterol from the active fractions, confirming its identity through Liquid Chromatography–Time-of-Flight Mass Spectrometry (LC-TOF-MS) and NMR analysis. When subjected to the same in vitro assay, stigmasterol demonstrated similar effects to GPCE and its active fractions, including HMGCR inhibition and attenuation of OA-induced lipid accumulation in hepatocytes. These in vitro findings are consistent with recent in vivo animal studies, where stigmasterol was shown to protect against high-fat and high-cholesterol diet-induced steatohepatitis [21] and MASLD in mouse models [19]. The GPCE (50 µg/mL) inhibited HMGCR by 86.31 ± 5.3%, whereas stigmasterol (5 µM) inhibited HMGCR activity by 56.59 ± 7.6%. Although exact quantitative profiling of stigmasterol content within the crude extract was not performed in this study, individual phytosterols generally represent a minor proportion of total crude plant extracts [26]. Therefore, stigmasterol identified in *G. procumbens* may represent one of the potential bioactive constituents that could contribute to its observed biological activities. Interestingly, despite exhibiting lower HMGCR inhibitory activity compared with GPCE under the tested conditions, stigmasterol demonstrated comparable attenuation of lipid accumulation in OA-induced HepG2 cells. Nevertheless, given the complex phytochemical composition of GPCE, the overall activity of the extract may be influenced by other phytochemicals reported in *G. procumbens* such as chlorogenic acid, kaempferol, and trans-*p*-coumaric acid.

To further explore the molecular changes associated with the anti-steatotic effect, we investigated the mRNA expression of key genes involved in cholesterol homeostasis. The active fractions and isolated stigmasterol significantly downregulated the mRNA expression of *SREBP2* and *PCSK9* mRNA expression in the OA-induced steatotic HepG2 model. Because alteration in cholesterol homeostasis has been implicated in MASLD pathogenesis, changes in the expression of these genes are biologically relevant. Previous research indicated that *G. procumbens* ameliorated ethanol-induced liver steatosis and was associated with downregulation of the SREBP-1c pathway, a key regulator in de novo lipogenesis [15]. Although a different steatosis model was employed, our findings suggest that the lipid regulatory effects of *G. procumbens* may also be associated with changes in cholesterol homeostasis-related gene expression under fatty-acid-induced steatotic conditions. Furthermore, the sustained downregulation of *SREBP2* mRNA expression by stigmasterol across all tested exposure durations (6, 12, and 24 h) is consistent with previous reports that phytosterols influence SREBP2 regulation, including reduced hepatic nuclear SREBP2 abundance in animal models [27]. Similarly, the continuous suppression of *PCSK9* expression further supports an effect of stigmasterol on cholesterol-related pathways. Interestingly, our study revealed distinct differences between stigmasterol and atorvastatin. While atorvastatin’s inhibitory effect on *SREBP2* diminished by 24 h, stigmasterol maintained significant suppression. Furthermore, stigmasterol induced time-dependent modulation of *HMGCR* mRNA expression without significantly altering *LDLR* expression, whereas atorvastatin caused varying temporal fluctuations in both genes. These findings suggest that stigmasterol may contribute to the lipid-lowering effects of *G. procumbens* and is associated with a distinct pattern of SREBP2-related gene expression compared with the compensatory transcriptional responses observed following atorvastatin treatment. Mechanistically, statins are known to reduce intracellular cholesterol, triggering SREBP2 and compensatory upregulation of PCSK9 and LDLR. While LDLR increases hepatic LDL uptake, PCSK9 counteracts this response by promoting LDLR degradation [28]. In contrast, phytosterols such as stigmasterol have been reported to influence cholesterol homeostasis through effects on sterol-regulated pathways [29]. These effects may provide a possible explanation for the observed suppression of *SREBP2* and *PCSK9* mRNA following stigmasterol treatment. However, the specific upstream regulatory mechanisms were not investigated in the present study and warrant further investigation.

While our findings provide novel insights into the biological activity and transcriptional effects of *G. procumbens* and stigmasterol, several limitations remain to be addressed. First, additional mechanistic studies would strengthen the understanding of the pathway involved. Specifically, protein-level validation of SREBP2, PCSK9, HMGCR and LDLR by Western blotting, together with functional assays such as LDL uptake and quantitative cellular cholesterol and triglyceride measurements, would provide complimentary evidence for the transcriptional findings. Additionally, assessing broader lipid metabolism markers related to de novo lipogenesis, such as fatty acid synthase and stearoyl-CoA desaturase, and fatty acid β-oxidation, such as carnitine palmitoyl transferase 1A, could further clarify the mechanism underlying the reduction in lipid accumulation beyond cholesterol-specific genes. Future studies could extend these findings to additional hepatocyte models, such as Huh7 and primary hepatocytes, to determine the reproducibility and physiological relevance of the observed effect. Further in vivo investigations are also warranted to evaluate the safety and efficacy of both *G. procumbens* extract and stigmasterol on liver histology and cardiometabolic endpoints in diet-induced MASLD models.

## 4. Materials and Methods

### 4.1. Plant Collection, Extraction

*G. procumbens* plants were collected from Taman Botani, Universiti Kebangsaan Malaysia (voucher specimen UKMB40343). Air-dried powdered leaves were extracted with 95% ethanol (1:20, *w*/*v*) by three successive 3-day macerations. Filtrates were concentrated using a rotary evaporator (<40 °C) at 100 rpm and freeze-dried to yield the crude extract (GPCE).

### 4.2. Fractionation, Purification and Isolation of Compound from GPCE

The GPCE was sequentially fractionated using hexane, ethyl acetate, and acetone to yield the hexane (GPHF), ethyl acetate (GPEA), and acetone (GPAF) fractions. Each fraction was subjected to silica gel column chromatography (silica gel 60, 230–400 mesh) using gradient elution systems of varying polarities (comprising hexane, ethyl acetate, acetone, chloroform and methanol). The hexane fraction was eluted with hexane:ethyl acetate (9:1, 8:2, 7:3, 6:4, 5:5), ethyl acetate 100%, hexane:acetone (9:1, 7:3, 1:1), chloroform 100%, chloroform:ethyl acetate (9:1, 7:3, 1:1), dichloromethane:ethyl acetate (9:1, 7:3, 1:1), ethyl acetate:methanol (5:5), and methanol 100%. The ethyl acetate fraction was eluted with hexane:ethyl acetate (7:3, 6:4, 5:5), ethyl acetate 100%, hexane:acetone (9:1, 7:3, 1:1), chloroform 100%, chloroform:ethyl acetate (9:1, 7:3, 1:1), dichloromethane:ethyl acetate (9:1, 7:3, 1:1), ethyl acetate–methanol (5:5), and methanol 100% and the acetone fraction with ethyl acetate 100%, chloroform:ethyl acetate (9:1, 7:3, 1:1), dichloromethane:ethyl acetate (9:1, 7:3, 1:1), ethyl acetate:methanol (5:5), and methanol 100%. Subfractions were monitored by thin-layer chromatography (TLC) and pooled according to their R_f_ values and further purified on a Sephadex LH-20 (dichloromethane:methanol, 4:1, *v*/*v*) and recrystallised (petroleum ether:methanol 9.5:0.5, *v*/*v*). Purity was assessed via TLC (hexane:ethyl acetate 9:1, *v*/*v*), visualised under UV (265 nm) and 5% H_2_SO_4_ spraying.

### 4.3. Characterisation and Identification of Stigmasterol (Compound ***1***)

Liquid Chromatography–Time-of-Flight Mass Spectrometry (LC–TOF–MS) was performed using an UltiMate 3000 UHPLC system (Dionex, Germering, Germany) equipped with an Acclaim™ Polar Advantage II C18 column (Thermo Fisher Scientific, CA, USA) (3 × 150 mm, 3 μm) at 40 °C, a flow rate of 0.4 mL/min, and an injection volume of 1 μL. Gradient elution employed H_2_O + 0.1% formic acid (A) and acetonitrile (B): 5% B (0–3 min), 80% B (3–10 min), held at 80% B (10–15 min), and 5% B (15–22 min). Mass detection was carried out on a MicroTOF-Q III mass spectrometer (Bruker Daltonics, Bremen, Germany) with ESI in positive ion mode (capillary 4500 V, nebulizer 2.0 bar, dry gas 8.0 U/min at 300 °C, scan range *m*/*z* 50–1500, end plate offset −500 V, collision cell RF 200 Vpp). Mass data were processed using Compass DataAnalysis software version 4.1. For NMR analysis, the purified compound was dissolved in CDCl_3_ and analysed using a Bruker Ascend 600 MHz CryoProbe spectrometer (Bruker Daltonics, Bremen, Germany) to record one-dimensional ^1^H and ^13^C NMR spectra, which were compared with reported data to confirm its identification as stigmasterol.

### 4.4. Preparation of GPCE, GPHF, GPEA, GPAF and Stigmasterol for Cell Treatment

GPCE, GPHF, GPEA, GPAF were dissolved in dimethyl sulfoxide (DMSO) (Sigma-Aldrich, St. Louis, MO, USA) to a 50 mg/mL stock, sonicated and sterile-filtered (0.22 µm, Merck, Darmstadt, Germany). The commercial stigmasterol (412.69 g/mol) was prepared as a 10 mM stock. All treatments were subsequently diluted in minimum essential media (MEM) containing 2% foetal bovine serum (FBS).

### 4.5. HMG-CoA Reductase Inhibition

The HMGCR inhibitory activity was determined using an HMG-CoA reductase inhibition assay kit (Sigma-Aldrich, St. Louis, MO, USA) following the manufacturer’s instructions. GPCE (50 µg/mL), fractions (25–50 µg/mL), and stigmasterol (2.5–5 µM) were incubated with NADPH, HMG-CoA substrate, and HMGCR enzyme at 37 °C. Absorbance was monitored at 340 nm using a microplate reader (PerkinElmer, Inc., Waltham, MA, USA) and recorded every 20 s for 10 min. The percent inhibition was calculated relative to the enzyme control using the kit-provided pravastatin as a positive control, whereas atorvastatin was utilised as the reference standard in all subsequent cell-based assays.

### 4.6. Cell Culture

HepG2 cells (ATCC HB-8065, American Type Culture Collection, Rockville, MD, USA) were cultured in MEM (Nacalai Tesque Inc., Kyoto, Japan) with 10% FBS (Tico, Bristol, UK) and 1% Penicillin–Streptomycin (Nacalai Tesque Inc., Kyoto, Japan). The cells were cultured in 75 cm^2^ flask and stored in the incubator at 37 °C in 5% CO_2_ (ESCO, Celculture CO_2_ incubator, Changi, Singapore). Changing the media of cells was carried out 24 h after culturing and then after 48 h until the cells were 90% confluent. At that confluency, subculturing of cells was carried out for multiple passage until the cells were enough for seeding and treatment.

### 4.7. Cell Viability Assay

HepG2 cells (1 × 10^4^ cells/well) were seeded in a 96-well plate for 24 h, followed by serum starvation for 6 h in medium containing 2% FBS. Cells were treated with GPCE, GPHF, GPEA, GPAF (6.25, 12.5, 25, 50, 100, 200 µg/mL) and stigmasterol (0.875, 1.25, 2.5, 5, and 10 μM) for 24 h. Following treatment, the medium was replaced with 90 μL serum-free medium and 10 μL 3-(4,5-dimethylthiazol-2-yl)-2,5-diphenyltetrazolium bromide (MTT) solution (final concentration 0.5 mg/mL) and incubated for 4 h at 37 °C in 5% CO_2_. The supernatant was removed, and formazan crystals were dissolved in 150 μL DMSO per well. Absorbance was measured at 570 nm using a microplate reader (NanoQuant, Tecan Group, Grödig, Austria). Percentage of cell viability was calculated using the equation (A_treated_/A_control_) × 100, where A_treated_ is the absorbance of treated cells and A_control_ is the absorbance of control cells.

### 4.8. Oil Red O Staining

HepG2 cells (1 × 10^5^ cells/well) were seeded in a 6-well plate overnight. Cells were pretreated with GPCE (50 µg/mL), GPHF (25 µg/mL), GPEA (50 µg/mL), and GPAF (50 µg/mL) for 12 h and stigmasterol for 6, 12 and 24 h, followed by induction with 0.5 mM oleic acid (Sigma Aldrich, St. Louis, MO, USA) for 24 h (adopted and modified from Tie et al. 2021 and Liu et al. 2024) [30,31]. OA was added from stock solutions such that the final DMSO concentration did not exceed 0.1%. The media was removed and then HepG2 cells were fixed with 10% neutral buffered formalin (Chemiz, Staffordshire, UK) for 30 min and stained with Oil Red O solution (Sigma Aldrich, St. Louis, MO, USA) for 20 min. The cells were washed with PBS and observed under a microscope (Olympus, Tokyo, Japan). For lipid quantification in HepG2 cells, 100% isopropanol was added to dissolve the Oil Red O reagent, and the absorbance was measured at 500 nm (NanoQuant, Tecan Group, Grödig, Austria). The percentage of lipid accumulation was calculated as A_treated_/A_control_ × 100 where A_treated_ is the absorbance of treated sample and A_control_ is the absorbance of the control cells.

### 4.9. Quantitative Real-Time Polymerase Chain Reaction (qRT-PCR) Analysis

HepG2 cells were treated with GPCE, GPHF, GPEA, GPAF and stigmasterol following the same pretreatment and OA induction protocol as used in the Oil Red O assay (Section 4.8). After treatment, total RNA was extracted using TRIzol reagent (Thermo Fisher, Waltham, MA, USA) according to the manufacturer’s instructions. cDNA was synthesised from 1 μg of DNase-treated RNA using a RevertAid First Strand cDNA synthesis kit (Thermo Fisher, Waltham, MA, USA). qRT-PCR was performed on a Bio-Rad CFX Real-Time PCR thermocycler (Bio-Rad Laboratories, Hercules, CA, USA) under standard cycling conditions (95 °C for 30 s, 40 cycles of 95 °C for 5 s and 60 °C for 30 s). Relative mRNA expression was calculated using the 2^−ΔΔCt^ method, normalised to glyceraldehyde-3-phosphate dehydrogenase (GAPDH) and TATA-binding protein (TBP), and all reactions were carried out in triplicate [32]. Primer specificity was confirmed by the presence of a single melt-curve peak and a single band of the expected size on agarose gel electrophoresis. Specific primer sequences of genes involved were as follows: *PCSK9*, 5′-CCAAGCCTCTTCTTACTTCACC-3′ and 5′-GCATCGTTCTGCCATCACT-3′; *SREBP2*, 5′-CTCTGACCAGCACCCACACT-3′ and 5′-CACACCATTTACCAGCCATAAG-3′; *HMGCR*, 5′-ATAACACGATGCATAGCCATCCTG-3′ and 5′-AAAATTGTGAAAAGGCCAGCAATAC-3′; *LDLR*, 5′-CTGAAATCGCCGTGTTACTG-3′ and 5′-GCCAATCCCTTGTGACATCT-3′

### 4.10. Statistical Analysis

Data generated were expressed as mean ± standard error of mean (SEM) of at least three independent experiments. Statistical analysis was performed using GraphPad Prism 9 (GraphPad Software, Inc., CA, USA). Differences between groups were analysed by one-way analysis of variance (ANOVA), followed by Dunnett’s multiple comparisons test, which compares each treatment group to a single control (e.g., normal control or OA-induced group). Cell-based experiments were performed with at least three biological replicates, whereas the HMG-CoA reductase inhibition assay was conducted with at least three independent technical replicates. A *p*-value of <0.05 was considered statistically significant.

## 5. Conclusions

In conclusion, *G. procumbens* extract exhibited potent HMG-CoA reductase inhibitory activity, reduced OA-induced lipid accumulation, and was associated with altered expression of selected cholesterol homeostasis-related genes in steatotic HepG2 cells. Bioassay-guided fractionation identified stigmasterol as one of the bioactive constituents contributing to these observed biological activities. Collectively, these findings support the potential of *G. procumbens* as a promising source of bioactive compounds for the management of MASLD and provide a basis for further mechanistic investigations involving protein-level validation, functional assays, and in vivo studies.

## Data Availability

The datasets generated and/or analysed during the current study are available from the corresponding author on reasonable request.

## Abbreviations

The following abbreviations are used in this manuscript:

DMSO	dimethyl sulfoxide
ESI	electrospray ionisation
FBS	foetal bovine serum
GAPDH	glyceraldehyde-3-phosphate dehydrogenase
GPCE	*G. procumbens* crude extract
GPHF	*G. procumbens* hexane fraction
GPEA	*G. procumbens* ethyl acetate fraction
GPAF	*G. procumbens* acetone fraction
HMGCR	3-hydroxy-3-methylglutaryl-CoA reductase
LC-TOF-MS	liquid chromatography–time-of-flight mass spectrometry
LDLR	low density lipoprotein receptor
MASH	metabolic dysfunction-associated steatohepatitis
MASLD	metabolic dysfunction-associated steatotic liver disease
MEM	minimum essential media
MTT	3-(4,5-dimethylthiazol-2-yl)-2,5-diphenyltetrazolium bromide.
NMR	nuclear magnetic resonance
OA	oleic acid
ORO	Oil Red O
PBS	phosphate-buffered saline
PCSK9	proprotein convertase subtilisin/kexin type 9
qRT-PCR	quantitative real-time polymerase chain reaction
SREBP2	sterol regulatory element-binding protein 2
TBP	TATA-binding protein
TLC	thin-layer chromatography
